# Public service motivation, public sector preference and employment of Kenyan medical doctor interns: a cross-sectional and prospective study

**DOI:** 10.1186/s12960-024-00945-6

**Published:** 2024-09-02

**Authors:** Daniel Mbuthia, Yingxi Zhao, David Gathara, Catia Nicodemo, Gerry McGivern, Jacinta Nzinga, Mike English

**Affiliations:** 1 KEMRI-Wellcome Trust Research Programme, Nairobi, Kenya; 2 NDM Centre for Global Health Research, Nuffield Department of Medicine, University of Oxford, S Parks Rd, Oxford, OX1 3SY UK; 3 MARCH Centre, London School of Hygiene and Tropical Medicine, London, UK; 4 Nuffield Department of Primary Care Health Sciences, University of Oxford, Oxford, UK; 5 Department of Economics, Verona University, Verona, Italy; 6 King’s Business School, King’s College London, London, UK

**Keywords:** Public sector, Public service motivation, Employment, Labour market

## Abstract

**Background:**

Kenya grapples with a paradox; severe public sector workforce shortages co-exist with rising unemployment among healthcare professionals. Medical schools have increased trainee outputs, but only 45% of newly qualified/registered doctors were absorbed by the public sector during 2015–2018. In such a context, we explore what influences doctors’ career choices at labour market entry, specifically understanding the role of public service motivation (PSM).

**Methods:**

We conducted a cross-sectional and prospective study of interns and recently graduated doctors to examine PSM, their intention to work in the public sector and their final employment sector and status. We surveyed them on their PSM and job intentions and conducted a prospective follow-up survey of the interns, around one year later, to understand their employment status.

**Findings:**

We recruited 356 baseline participants and followed up 76 out of 129 eligible interns. The overall PSM score was high among all participants (rated 4.50/5.00) irrespective of sector preferences. 48% (171/356) of the participants preferred to work in the public sector immediately after internship, alongside 16% (57/356) preferring direct entry into specialist training—commonly in the public sector. Only 13% (46/356) and 7% (25/365) preferred to work in the private or faith-based sector. Despite the high proportion of interns preferring public sector jobs, only 17% (13/76) were employed in the public sector at follow-up and 13% (10/76) were unemployed, due to lack of job availability.

**Conclusion:**

High PSM scores irrespective of sector preferences suggest that doctors are generally committed to serving the ‘public good’. Many intended to work in the public sector but were unable to due to lack of job opportunities. Policymakers have an opportunity to tackle workforce gaps in the public sector as young doctors continue to express a preference for such work. To do this they should prioritise creating adequate and sustainable job opportunities.

**Supplementary Information:**

The online version contains supplementary material available at 10.1186/s12960-024-00945-6.

## Introduction

Public administration scholars have long been inquiring about what motivates people to join/work for the public sector and the value of this motivation to public organisations. Perry and Wise (1990) introduced the concept of public service motivation (PSM), which they defined as “an individual’s predisposition to respond to motives grounded primarily or uniquely in public institutions and organizations” [1]. Perry also later developed a PSM measure from a US population sample which includes four conceptual dimensions of PSM: attraction to public policy making, commitment to public interest, compassion, and self-sacrifice [2]. The critique of Perry’s scale over its cross-cultural and international application led to a revision of the scale by Kim and colleagues, who adapted the PSM dimensions and the measure to make it suitable across diverse cultures and languages [3].

Work examining PSM has consistently demonstrated the consequences of PSM in relationship to person–organisation fit, job satisfaction, commitment [4–6], and retention. Studies have also focused on whether PSM is associated with individuals’ sector choices, and whether PSM could form the basis of recruiting candidates for public organisations [7, 8]. A number of studies have established that measures of PSM among public servants are greater than for those working in private organisations [6, 9]. Other studies have examined whether PSM exists prior to job entry and whether it could predict public sector preference [4, 9–12]. However, PSM has only been studied in a narrow range of national contexts, mostly in high-income countries, and very few studies have used Kim’s PSM scale, especially in low- and middle-income country (LMIC) settings. Our study therefore extends PSM research by using Kim’s scale, and investigating its association with the career decisions of Kenyan medical doctors who are in their internship and expected, after one year, to transition to independent clinical practice as fully licensed medical practitioners. We suggest this pre-licensure phase is critical in shaping individuals’ career trajectories, and of great interest considering the workforce shortages of medical doctors, especially in the public sector in LMIC contexts.

### The Kenyan context

The public sector provides the largest share of healthcare in Kenya. Approximately 46% of health facilities are run by the government in 2022, 44% by the private sector and the rest by faith-based and non-governmental organisations [13]. However, the public sector provides a greater proportion of inpatient hospital care, particularly to the poor and those in rural areas [14], as the costs of utilising public hospital services are lower. Additionally, a large number of private sector facilities are small clinics and dispensaries [15].

Kenya grapples with a shortage of medical workforce, with an estimated density of 0.23 per 1000 population in 2020. This is significantly lower than the average for LMICs (1.4 per 1000 in 2017) [16]. Medical doctors are typically trained in universities through a Bachelor of Medicine & Bachelor of Surgery (MBChB) programme that lasts six years with an additional internship year. As with other African countries, medical schools in Kenya are expanding to meet health workforce gaps. Now 11 medical schools are providing undergraduate medical training with a combined annual output of 628 in 2019 [17], nearly double that of only a few years previously (287 in 2006, 320 in 2016) [18–20].

Before 2013, when Kenya devolved responsibility for health systems to 47 semi-autonomous county governments, medical doctors were automatically employed and deployed by the national government after their internship. Since devolution, counties have managed health workforce recruitment and management [21, 22], and automatic employment of medical doctors post-internship ended. This means that medical doctors now must apply for jobs after internship, but whether they are employed is contingent on vacancies advertised by county/national government(s) and other organisations. Most doctors who completed their internship would initially be employed as medical doctors in public, private or faith-based health facilities. Public sector roles in the Ministry of Health or County Health Department or parastatal organisations typically require some prior working experience with entry into specialist training also requiring 2–3 years’ work experience.

Recent data from the Kenyan Ministry of Health suggested between 2015 and 2018, only 45% (825/1,800) of newly qualified and registered doctors were employed in the public sector [19]. Such low absorption could be due to the lack of job opportunities provided by governments (“demand side”) [23], or medical doctors’ preferring alternatives to public sector jobs (“supply side”), potentially due to the poor conditions they face as interns in public hospitals [24]. Understanding what influences medical doctors’ employment choices is crucial for shaping effective healthcare policies and workforce planning, especially as the majority of hospital care provided to the population is still through the public sector [14]. Several studies in Kenya have investigated healthcare professionals’ public sector preferences [25, 26]. However, to the best of our knowledge, no previous study has examined the concept of PSM and its relations with Kenyan medical interns’ career preferences. Our study fills this gap.

## Methods

We used a combined cross-sectional and a prospective study design to examine PSM, intention to work in the public sector and final employment sector among medical interns and recently graduated medical doctors in Kenya. This study is embedded in a larger project that focuses on the internship training experiences of Kenyan doctors [24].

From Nov 2021 to May 2022, we conducted a cross-sectional survey of Kenyan medical interns and medical doctors who completed their internship within 3 years. We used a mix of convenience and snowball sampling approaches [27] to include survey participants. We worked with different stakeholders and asked them to share the survey through their respective platforms such as WhatsApp or short message service (SMS). Our approach included working with (a) the Kenyan Medical Practitioners and Dentists Council, which shared the survey with all eligible medical officers on their registry through SMS; (b) the Kenya Medical Association Young Doctor Network and Kenya Young Doctor Caucus that shared within their respective WhatsApp groups; (c) three major medical schools based in or near Nairobi that shared within recent graduate class representative; and (d) selected facilities in the Clinical Information Network operated by KEMRI-Wellcome Trust Research Programme [28, 29], which shared the survey within facilities’ own WhatsApp groups. The survey was online and self-administered through REDCap, and one GB of data (worth 250 KES or 1.8 GBP) were given to respondents who fully completed the survey. Out of an estimated 2,400 eligible participants (estimated 600 graduates per year), 498 started the survey and 356 fully completed the survey and so were included for analysis. Out of the 356 sample, 227 were medical doctors who completed their internship within 3 years and 129 were current interns.

The cross-sectional survey questionnaire focuses on PSM and public sector intention. It includes questions on (1) demographics and medical training (name of medical school and internship hospital as well as funding for medical training); (2) preferred job roles immediately after internship and five years after internship (one of seven options listed in Table 3, or others). Medical doctors who had already completed internship and were already in the labour market were required to indicate the year of internship completion, and what was their most preferred option when they finished internship; and (3) PSM evaluation using the scale developed by Kim et al. [3]. The scale has 16 items grouped into four factors and domains, i.e. attraction to public participation, commitment to public values, compassion, and self-sacrifice. The questionnaire was developed as part of a broader medical internship experience project and questions were pre-tested including the use of cognitive interviews [30, 31] to ensure that all questions were relevant and that respondents could fully understand the questions.

We further conducted a prospective telephone follow-up of the 129 medical interns from March to May 2023, around one year later, to understand their final employment status. We only followed up participants who had consented to a follow-up interview at baseline, and who provided us with their phone numbers. The follow-up survey included five questions on their current role, locations, employment terms, and also an open-ended question on reasons for current employment.

Data were managed and analysed using STATA (Stata V.17). Descriptive statistics on frequencies, percentages and means were used to describe the demographics, PSM and public sector intention of all baseline participants as well as and final employment status of follow-up participants. For example, we calculated the mean score (out of 5) across four PSM domains and the aggregated PSM score. We also conducted a confirmatory factor analysis to examine the performance of PSM in the sample and the degree to which items are related to the underlying dimension as proposed by Kim et al. [3].

Ethical approval for the study is issued by Oxford Tropical Research Ethics Committee (OxTREC 563-20 and OxTREC 518-21) and Kenya Medical Research Institute (KEMRI) (SERU 4071). Electronic consents or written consents were obtained for all survey participants.

## Results

### Participant characteristics

Basic sociodemographic and medical training characteristics of baseline participants are presented in Table 1. 36% (129/356) of the survey population were interns at the time of the survey, having completed 6.5 months of internship training on average; the rest were post-internship doctors that mostly completed their internship from 2020 to 2022. The University of Nairobi contributed to 57% (201/356) of the survey population, it is also the largest medical school in Kenya graduating nearly half of the doctors. Most participants undertook their internship in public facilities (78%, 276/356), whereas 73% of internship training centres are public hospitals [32]. As for follow-up, 116 out of 129 interns agreed to be followed up through telephone calls. We successfully followed up 77 of them with complete data (66%). The time from baseline to follow-up is on average 14 months.

**Table 1:** Characteristics of the Kenya survey population

	Medical intern (*N* = 129)	Medical doctor (*N* = 227)	Total (*N* = 356)
Gender (*n*, %)			
Male	74 (57.4%)	108 (47.6%)	182 (51.1%)
Female	55 (42.6%)	114 (50.2%)	169 (47.5%)
Others/prefer not to say	0	5 (2.2%)	5 (1.4%)
Age (mean, SD)	27.6 (2.2)	28.9 (2.1)	28.4 (2.3)
Marital status (*n*, %)			
Single	118 (91.5%)	169 (74.5%)	287 (80.6%)
Married	11 (8.5%)	54 (23.8%)	65 (18.3%)
Divorced, widowed and separated	0	4 (1.7%)	4 (1.2%)
Months of internship training completed (for intern only, mean, SD)	6.5 (3.1)	–	
Year of completing internship (for medical doctor only, *n*, %)			
2022	–	52 (22.9%)	
2021	–	54 (23.8%)	
2020	–	60 (26.4%)	
2019	–	42 (18.5%)	
2018	–	19 (8.4%)	
Medical school graduated (*n*, %)			
University of Nairobi	63 (48.8%)	138 (60.8%)	201 (56.5%)
Kenyatta University	31 (24.0%)	21 (9.3%)	52 (14.6%)
Kenya Methodist University	2 (1.6%)	18 (7.9%)	20 (5.6%)
Maseno University	6 (4.7%)	19 (8.4%)	25 (7.0%)
Jomo Kenyatta University	16 (12.4%)	9 (4.0%)	25 (7.0%)
Others	11 (8.5%)	22 (9.7%)	35 (9.8%)
Funding for medical school (*n*, %)			
Completely self-funded	58 (45.0%)	117 (51.5%)	175 (49.2%)
Received full or partial government scholarship	66 (51.2%)	104 (45.8%)	170 (47.8%)
Others	5 (3.8%)	6 (2.6%)	11 (3.1%)
Internship hospital ownership (*n*, %)			
Public	100 (77.5%)	176 (77.5%)	276 (77.5%)
Private	1 (0.8%)	2 (0.9%)	3 (0.8%)
Mission	17 (13.2%)	36 (15.9%)	53 (14.9%)
Others	11 (8.5%)	13 (5.7%)	24 (6.7%)

### Public service motivation

As shown in Table 2 in the item- and factor-specific means. Self-sacrifice, which is the foundational concept representing the altruistic or pro-social origins of PSM, was rated slightly lower in our sample (factor mean 3.88 out of 5). For example, Q16 “I would agree to a good plan to make a better life for the poor, even if it costs me money” was rated 4.04 out of 5. Nonetheless, despite overall lower average compared to other domains, some factors under self-sacrifice, i.e. Q13 “I am prepared to make sacrifices for the good of society” were rated quite highly indicating substantial commitment and inherent value to serving public good being integral to medical doctors’ professional identity and could therefore explain the overall high PSM among medical doctors. Other factors and the overall PSM score (4.50 out of 5) were relatively high, suggesting that most respondents had high motivation for public service. Also shown in Additional fle 1, older participants and those who received scholarships during medical training had higher PSM scores.

**Table 2:** Performance of Kim’s public service motivation scale

Factor	No.	Item	Mean (SD)	Domain mean (SD)	Cronbach’s alpha
Attraction to public participation	Q1	I admire people who initiate or are involved in activities to aid my community	4.71 (0.55)	4.68 (0.49)	0.8754
	Q2	It is important to contribute to activities that tackle social problems	4.73 (0.47)		
	Q3	Meaningful public service is very important to me	4.65 (0.66)		
	Q4	It is important for me to contribute to the common good	4.64 (0.61)		
Commitment to public values	Q5	I think equal opportunities for citizens are very important	4.77 (0.46)	4.75 (0.41)	0.7601
	Q6	It is important that citizens can rely on the continuous provision of public services	4.69 (0.61)		
	Q7	It is fundamental that the interests of future generations are taken into account when developing public policies	4.78 (0.49)		
	Q8	To act ethically is essential for public servants	4.78 (0.55)		
Compassion	Q9	I feel sympathetic to the plight of the underprivileged	4.64 (0.70)	4.67 (0.47)	0.7745
	Q10	I empathise with other people who face difficulties	4.68 (0.57)		
	Q11	I get very upset when I see other people being treated unfairly	4.67 (0.61)		
	Q12	Considering the welfare of others is very important	4.72 (0.52)		
Self-sacrifice	Q13	I am prepared to make sacrifices for the good of society	4.22 (0.91)	3.88 (0.87)	0.8365
	Q14	I believe in putting civic duty before self	3.94 (1.08)		
	Q15	I am willing to risk personal loss to help society	3.32 (1.27)		
	Q16	I would agree to a good plan to make a better life for the poor, even if it costs me money	4.04 (0.95)		
Overall				4.50 (0.43)	0.8780

All items were responded to on a 5-point Likert-type scale, where 1 refers to strongly disagree and 5 refers to strongly agree

Indices of the PSM developed by Kim et al. [3], which has not been previously tested in the Kenyan sample, showed a moderate fit according to the confirmatory factor analysis (comparative fit index [CFI] = 0.92, root mean square error of approximation [RMSEA] = 0.08, standardised root mean squared residual [SRMR] = 0.06). When removing item Q2, as Kim and colleagues suggest [33], there was a slight improvement in RMSEA but still a moderate fit. The reliability coefficient (Cronbach’s alpha) for the overall scale was 0.88 and the coefficients for the four factors ranged from 0.76 to 0.88 (Table 2).

### Intention to work in the public sector

Baseline participants (*n* = 356) were asked about their preferred job roles immediately after internship and five years after internship (Table 3). Most participants (48%, 171/356) stated a preference to work in the public sector as medical doctors immediately after internship, followed by direct entry into specialist training (16%, 57/356)—which is also predominantly undertaken in the public sector. Preference for entry into the private sector (13%, 46/356) or faith-based sector (7%, 27/356) immediately after internship were low. As for job sector preference five years after internship, low preference for the private and faith-based sectors (10%, 36/356 and 4%, 14/356) persisted. At 5 years slightly fewer respondents intended to remain working in the public sector (21%, 75/356) and more stated a preference to go into specialist medical training (42%, 150/356). These trends are similar between the 129 interns and the post-internship doctors (Table 4).

**Table 3:** Career intention for baseline participants (*n* = 356)

	All participants (*n* = 356)	
	Intention immediately after internship (%)	Intention five years after internship (%)
Public sector national/county hospitals (as medical doctor)	48.0	20.5
Faith-based not-for-profit hospitals or clinics (as medical doctor)	6.5	3.9
Private-for-profit hospitals or clinics (as medical doctor)	12.9	10.1
Public sector role in the Ministry of Health/County Health Department or parastatal organisations	6.2	8.7
Not-for-profit technical assistance organisations (public health/health management/health programme)	2.8	5.1
Research organisation or research training	7.3	8.7
Specialist medical training (MMed)	15.5	41.6
Others	0.8	1.4

**Table 4:** Comparison between career intention at the end of internship and final employment for medical officer interns around one year after internship

	Intern participants at baseline (*n* = 129)	Intern participants with follow-up data (*n* = 76)	
	Baseline intention immediately after internship (%)	Baseline intention immediately after internship (%)	Follow-up employment around one year after internship (%)
Public sector national/county hospitals (as medical doctor)	44.2	48.7	17.3
Faith-based not-for-profit clinical service organisations (as medical doctor)	4.7	4.0	10.7
Private-for-profit hospitals or clinics (as medical doctor)	10.1	9.2	54.7
Public sector role in the Ministry of Health/County Health Department or parastatal organisations	9.3	9.2	0
Not-for-profit technical assistance organisations (public health/health management/health programme)	2.3	2.6	0
Research organisation or research training	10.9	9.2	1.3
Specialist medical training (MMed)	17.8	15.8	0
Unemployed	N/A	N/A	13.3
Others	0.8	1.3	2.7

Intern participants at baseline (*n* = 129) including those who were lost to follow-up

We also compared PSM scores between baseline participants with different career intentions (Additional File 2). We found that participants with different intentions for career sector all had high PSM with minimal variations. For example, those who preferred to work in the public sector as medical doctors had an average PSM score of 4.45 out of 5, whereas those preferring to work in the private sector and faith-based sector had scores of 4.53 and 4.70 out of 5, respectively. Therefore, our findings suggest that PSM is not a useful discriminator for career intention, but instead other factors such as pay and working arrangement might influence career intention more.

### Employment in the public sector at follow-up

Table 4 presents the career intention and final employment of medical interns. Out of 129 interns recruited at baseline, we successfully followed up 76 participants who completed their internship and hereby focused on these participants. While 49% (37/76) of these participants indicated intention to work in the public sector as medical doctors at baseline, only 17% (13/76) indicated actual employment in the public sector one year after internship. Most respondents (65%, 49/76) ended up working in the private sector or faith-based hospitals despite not being the preferred job choice. Around one year after internship, no respondents were employed in the Ministry of Health/County Health Department or parastatal organisations, not-for-profit technical assistance organisations or enrolled in specialist trainings.

Additionally, 13% (10/76) of respondents suggested that they were unemployed at the time of follow-up. Of those who were employed, only 8% (5/66) were employed on a permanent contract, and more commonly they were employed on contract (52%, 34/66) or on a locum basis (41%, 27/66) especially for private-sector jobs. When asked reasons for choosing their current employment as an open-ended question, nearly 60% of respondents stated that these were the only available jobs for them, oftentimes only temporary job contracts available in private sectors.

## Discussion

Our cross-sectional and prospective study of Kenyan medical doctors and interns provided insights into their PSM, intention to work for the public sector and their final employment. Our findings show that the majority of the respondents generally had high motivation for public service. Most (48%) respondents stated a preference to work as medical doctors in the public sector, rather than the private or faith-based sector immediately after internship, but only 17% indicated actual employment in the public sector at one-year follow-up, with 65% working in private health care, and 13% unemployed.

There were also minimal variations between their PSM scores, which suggests that public sector job preference is not linked to PSM. We found that while all the other items/domains of PSM were rated high, the pro-social aspect of PSM, i.e. self-sacrifice, was rated relatively lower among the respondents. These findings are similar to Harris et al.’s study of Ugandan public servants, where Kim et al.’s scale suggested an overall high public service, commitment to public values, and compassion, and low willingness to self-sacrifice in favour of the common good [34]. Some studies reported that self-sacrifice is the most impactful and stable PSM dimension and is associated with a preference for public sector employment [35]. However, a recent review of PSM literature in Africa highlighted contradictory findings regarding the linkage between self-sacrifice and inclination towards public service, highlighting the possible differences in culture and value that could explain PSM and motives [6].

The minimal variations of PSM between participants with different career intentions also suggested that PSM is measuring a set of attitudes towards ‘public service’, not necessarily ‘public sector’ [36]. The concept of public sector could be interpreted differently in different cultures and settings [37]. In the Kenyan setting, working in faith-based not-for-profit organisations could also be considered as working for the ‘semi-public sector’. Because a medical doctor’s job is serving people and saving lives, regardless of the sectors they work, all respondents may believe they were working in the public interest and helping society. Our findings therefore indicated that PSM might not be the primary reason for the low absorption of medical doctors into the public sector in the Kenyan setting.

As noted, nearly half of the medical doctors surveyed indicated intention to work in public hospitals immediately after internship, but only 17% were employed in the public sector at follow-up. This finding is similar to previous qualitative work with medical doctors in Kenya and Uganda [23, 38]. The preference for the public sector could be due to various reasons beyond PSM and ‘serving the public’. For example, this may be due to better pay and job security, the public sector being “easier to work in” or linked enabling with dual practices, and culturally ingrained appreciation for public sector jobs [38–40]. In comparison, jobs in the private sector were perceived to have poorer contractual terms and involve higher workloads in the Kenyan setting. This contrasts with high-income settings, where private medical work is better paid [23, 38].

Kenya has boosted its supply of medical professionals through the expansion of medical training, although this has still not yet reached the country’s goal listed under its norms and standards [20]. However, demand for public sector doctors has increased more slowly, despite a shortage of doctors in the public sector. This is mostly due to the lack of sustained financial commitments for the health workforce and preference for other healthcare cadres [23]. Consequently, despite public sector preference many doctors remain unemployed, underemployed, or work in the private sector, often on temporary contracts, [23, 38]. Many doctors “just want a job anywhere (they) could find”, even if it is not the role they would have ideally chosen [38].

The findings of this study have implications for policy and practice. First on the positive side, our data support evidence that, for certain professions, the nature of the job serves some public good and therefore is perceived as ‘public service’, as evidenced by the high PSM scores regardless of sector preference. Given this, policymakers and healthcare managers therefore need to worry less about medical doctors’ public service values and motivations, but need to remain attentive to other factors that might influence doctors' career choices, satisfaction and retention.

Second, considering the mismatch between public sector intention and employment of doctors, and the high level of health worker unemployment, Kenya needs to prioritise workforce planning. This proactive approach could ensure that the training and development of medical doctors is aligned with national healthcare needs and employment capacities, therefore reducing the risk of unemployment among newly trained doctors. Additionally, many African countries have been conducting health labour market analysis [20, 41] and there exists an opportunity to integrate PSM into this analysis to better contextualise planning efforts as well as inform recruitment, retention, and motivation of healthcare workers. Significantly, LMICs need to re-think the causes of health workforce shortages in the public sector, as many are witnessing growing health workforce underutilisation and unemployment [42]. More in-depth, country-specific research evidence on the supply/demand mismatch dilemma is needed to draw policymakers’ attention to this issue.

Finally, our study adds to the global PSM literature by first using the PSM scale developed by Kim et al. in the Kenyan healthcare setting [3]. While Kim et al.’s scale was developed in 2013, with an international sample across 12 countries in response to criticisms of the American focus of the scale developed by Perry in 1996 [2], the majority of PSM scholarship still largely used the Perry scale when measuring PSM [4, 6, 43]. The use of Kim et al.’s scale is still limited in LMICs especially African countries, and many studies did not report on its psychometric performances. One exception is the work by Mikkelsen and colleagues which tested Kim et al. scale in four world regions including three countries in Africa (Ghana, Malawi and Uganda). Mikkelsen and colleagues suggested the scale’s partial metric invariance and scale non-invariance, i.e. causes and consequences of PSM are comparable across most countries but not means and its dimension. Our study suggested a moderate-to-good fit according to the confirmatory factor analysis and reliability coefficient, future studies should consider testing the scale’s performance in different populations and contexts.

### Limitations

There are some limitations that should be considered when interpreting our results. First, this work was conducted during the Covid-19 pandemic, which presented logistics challenges for data collection. We therefore used a mix of convenience and snowballing approaches to sample baseline respondents. The 356 responses came from all medical schools and 65 out of 74 internship hospitals. The sample is roughly 15% of the eligible population but not representative. Covid-19 might have also impacted the broad labour market dynamic. For example, high PSM among study participants and financial constraints of the government.

Second, at baseline, we included respondents who were interns and also those who already completed internships. While data in Appendix 1 suggested no significant differences between these participants, we acknowledge that responses from post-internship medical doctors could be subject to recall biases. Furthermore, we also did not survey these graduated doctors of their current sector of employment, and further comparing PSM scores between doctors in different sectors could help us further understand the role of PSM.

Third, while the risk of loss to follow-up is low considering the similar trends of job intention between baseline and follow-up intern respondents (Table 4), it is possible that people who did not respond to our baseline survey had different career intentions and employment outcomes. Moreover, we were only able to follow up interns on average 14 months after internship, and their career outcomes may be changing due to the emerging labour market dynamic in Kenya. Nonetheless, we still believe it provided a good description of the medical doctor and intern population.

Finally, we also acknowledge our self-administered survey, including PSM could be influenced by social desirability biases. This might be true for the relatively lower self-sacrifice domain. Considering the possible differences in culture and value that explain PSM but also respondents’ response patterns, future studies should continue testing PSM in diverse populations and contexts.

## Conclusion

We investigated PSM, public sector employment intention, and the actual employment of Kenyan medical doctors and interns. Our study found high scores of PSM among doctors irrespective of their sector preferences. These findings indicated an innate desire to serve the public good as a common trait among medical doctors in Kenya. PSM was not necessarily linked with sector preference due to contextual factors such as job stability and better pay which attracted medical doctors to public sector jobs. Many doctors stated their intention to work in the public sector but few were able to do so, due to a lack of job opportunities. Policymakers should focus on creating job opportunities to ensure medical doctors are recruited and retained in the public sector.

### Supplementary Information

Supplementary Material 1. Correlation analysis between PSM scores and other characteristics.

Supplementary Material 2. Comparison of PSM scores between different career intentions.

## Data Availability

The datasets generated during and/or analysed during the current study are available from the corresponding author on reasonable request.
