# Supplementary material for: Public service motivation, public sector preference and employment of Kenyan medical doctor interns: a cross-sectional and prospective study
Source: Hum Resour Health. 2024 Sep 2;22:61. doi: 10.1186/s12960-024-00945-6 (PMC11370027; doi:10.1186/s12960-024-00945-6)
Supplement: Supplementary file 1 — Supplementary Material 1. Correlation analysis between PSM scores and other characteristics. [file 12960_2024_945_MOESM1_ESM.docx]

**Appendix table 1.*Correlation analysis between PSM scores and other characteristics***

|  | PSM | APS | CPV | COM | SS | Female | Age | Single | Larger hospital | Received scholarship | Current MO |
| --- | --- | --- | --- | --- | --- | --- | --- | --- | --- | --- | --- |
| PSM | 1.00 |  |  |  |  |  |  |  |  |  |  |
| APS | 0.78* | 1.00 |  |  |  |  |  |  |  |  |  |
| CPV | 0.72* | 0.57* | 1.00 |  |  |  |  |  |  |  |  |
| COM | 0.77* | 0.53* | 0.66* | 1.00 |  |  |  |  |  |  |  |
| SS | 0.80* | 0.43* | 0.28* | 0.38* | 1.00 |  |  |  |  |  |  |
| Female | -0.02 | -0.04 | 0.01 | 0.09 | -0.07 | 1.00 |  |  |  |  |  |
| Age | 0.14* | 0.13* | 0.12* | 0.09 | 0.10 | -0.07 | 1.00 |  |  |  |  |
| Single | -0.05 | -0.05 | 0.02 | -0.03 | -0.07 | -0.06 | -0.31* | 1.00 |  |  |  |
| Larger hospital | 0.04 | 0.09 | -0.04 | -0.02 | 0.06 | 0.05 | 0.02 | -0.02 | 1.00 |  |  |
| Received scholarship | 0.12* | 0.10* | 0.05 | 0.04 | 0.14* | -0.33* | -0.85 | -0.11* | -0.06 | 1.00 |  |
| Current MO | 0.06 | 0.06 | 0.08 | 0.09 | -0.01 | 0.08 | 0.28* | -0.21* | 0.02 | -0.05 | 1.00 |
