# Supplementary material for: Public service motivation, public sector preference and employment of Kenyan medical doctor interns: a cross-sectional and prospective study
Source: Hum Resour Health. 2024 Sep 2;22:61. doi: 10.1186/s12960-024-00945-6 (PMC11370027; doi:10.1186/s12960-024-00945-6)
Supplement: Supplementary file 2 — Supplementary Material 2. Comparison of PSM scores between different career intentions. [file 12960_2024_945_MOESM2_ESM.docx]

**Appendix table 2.*Comparison of PSM scores between different career intentions***

| Immediately after internship (n=356) | PSM score | APS | CPV | COM | SS |
| --- | --- | --- | --- | --- | --- |
| ALL | 4.50 (0.43) | 4.68 (0.49) | 4.75 (0.41) | 4.67 (0.47) | 3.88 (0.87) |
| 1=Public sector national/county hospitals (as medical doctor) | 4.45 (0.47) | 4.63 (0.54) | 4.71 (0.43) | 4.60 (0.53) | 3.87 (0.87) |
| 2=Faith-based not-for-profit clinical service organizations (as medical doctor) | 4.70 (0.33) | 4.84 (0.35) | 4.90 (0.29) | 4.83 (0.33) | 4.24 (0.74) |
| 3=Private-for-profit hospitals or clinics (as medical doctor) | 4.53 (0.36) | 4.73 (0.38) | 4.73 (0.42) | 4.78 (0.33) | 3.88 (0.90) |
| 4=Public sector role in the Ministry of Health/County Health Department or parastatal organizations | 4.52 (0.37) | 4.72 (0.41) | 4.67 (0.47) | 4.70 (0.40) | 3.99 (0.72) |
| 5=Not-for profit technical assistance organizations (public health/health management/health program) | 4.61 (0.36) | 4.90 (0.32) | 4.95 (0.11) | 4.78 (0.34) | 3.83 (0.99) |
| 6=Research organization or research training | 4.39 (0.54) | 4.52 (0.60) | 4.67 (0.50) | 4.63 (0.54) | 3.76 (1.02) |
| 7=Specialist medical training (MMed) | 4.56 (0.35) | 4.75 (0.45) | 4.87 (0.27) | 4.75 (0.38) | 3.85 (0.83) |
| 8=Others | 4.40 (0.16) | 4.92 (0.14) | 4.83 (0.14) | 4.67 (0.38) | 3.17 (0.95) |
